# Microtissues Enhance Smooth Muscle Differentiation and Cell Viability of hADSCs for Three Dimensional Bioprinting

**DOI:** 10.3389/fphys.2017.00534

**Published:** 2017-07-25

**Authors:** Jin Yipeng, Xu Yongde, Wu Yuanyi, Sun Jilei, Guo Jiaxiang, Gao Jiangping, Yang Yong

**Affiliations:** ^1^Department of Urology, Chinese PLA General Hospital Beijing, China; ^2^Department of Urology, First Affiliated Hospital of Chinese PLA General Hospital Beijing, China

**Keywords:** human adipose derived stem cells, microtissues, smooth muscle differentiation, 3D bioprinting, tissue engineering, cell viability

## Abstract

Smooth muscle differentiated human adipose derived stem cells (hADSCs) provide a crucial stem cell source for urinary tissue engineering, but the induction of hADSCs for smooth muscle differentiation still has several issues to overcome, including a relatively long induction time and equipment dependence, which limits access to abundant stem cells within a short period of time for further application. Three-dimensional (3D) bioprinting holds great promise in regenerative medicine due to its controllable construction of a designed 3D structure. When evenly mixed with bioink, stem cells can be spatially distributed within a bioprinted 3D structure, thus avoiding drawbacks such as, stem cell detachment in a conventional cell-scaffold strategy. Notwithstanding the advantages mentioned above, cell viability is often compromised during 3D bioprinting, which is often due to pressure during the bioprinting process. The objective of our study was to improve the efficiency of hADSC smooth muscle differentiation and cell viability of a 3D bioprinted structure. Here, we employed the hanging-drop method to generate hADSC microtissues in a smooth muscle inductive medium containing human transforming growth factor β1 and bioprinted the induced microtissues onto a 3D structure. After 3 days of smooth muscle induction, the expression of α-smooth muscle actin and smoothelin was higher in microtissues than in their counterpart monolayer cultured hADSCs, as confirmed by immunofluorescence and western blotting analysis. The semi-quantitative assay showed that the expression of α-smooth muscle actin (α-SMA) was 0.218 ± 0.077 in MTs and 0.082 ± 0.007 in Controls; smoothelin expression was 0.319 ± 0.02 in MTs and 0.178 ± 0.06 in Controls. Induced MTs maintained their phenotype after the bioprinting process. Live/dead and cell count kit 8 assays showed that cell viability and cell proliferation in the 3D structure printed with microtissues were higher at all time points compared to the conventional single-cell bioprinting strategy (mean cell viability was 88.16 ± 3.98 vs. 61.76 ± 15% for microtissues and single-cells, respectively). These results provide a novel way to enhance the smooth muscle differentiation of hADSCs and a simple method to maintain better cell viability in 3D bioprinting.

## Introduction

Human adipose derived stem cells (hADSCs) are known for their multilineage differentiation potential, including bone, cartilage, adipose tissue, and smooth muscle (Bajek et al., 2016). Compared to mesenchymal stem cells (MSCs) derived from bone marrow or umbilical cords, hADSCs are easily accessible and abundant. Therefore, hADSCs have become an attractive stem cell source for tissue engineering. Many researchers have investigated the smooth muscle differentiation of hADSCs due to their promising applications in the field of cellular therapies involving urinary and cardiovascular systems (Choi et al., 2010; Fu et al., 2010; Salemi et al., 2015). These studies provided various methods for smooth muscle cell differentiation, including co-culturing hADSCs with primary myoblasts using heparin, 5-azacytidine or transforming growth factor β1 (TGF-β1) as smooth muscle inducing factors and attaching hADSCs to microcarriers created by thermally induced phase separation (TIPS; Di Rocco et al., 2006; Rodriguez et al., 2006; Meligy et al., 2012; Park et al., 2013; Parmar et al., 2015). Recent studies showed that growing hADSCs in a three-dimensional (3D) environment could strengthen their stemness and hADSCs could form 3D spheroids spontaneously (Bogdanova-Jatniece et al., 2014; Mineda et al., 2015; Shearier et al., 2016). These findings indicate that the 3D environment may accelerate the smooth muscle differentiation process of hADSCs through enhanced stemness. In a previous study, we formed microtissues (MTs) consisting of hADSCs via the hanging-drop method and inspected the internal framework of MTs by H&E staining. The results confirmed that the expression of vascular endothelial growth factor (VEGF) and Wnt5a were significantly higher in MTs than in hADSCs in adherent cultures (Xu et al., 2014). Thus, the aim of our present study is to explore a simple and efficient method of inducing hADSCs to differentiate into smooth muscle cells. We performed a novel inducement procedure by combining inducing factor TGF-β1 with the hanging-drop method to induce smooth muscle differentiation while generating hADSC microtissues.

Three dimensional bioprinting (3D bioprinting) is a new additive manufacturing tool in tissue engineering that can produce tissue with the assistance of designing software. Compared to conventional tissue engineering approaches, it can distribute cells spatially in the bioprinted tissues and control their architecture under physiological conditions, thus mimicking the natural state of tissues. However, cell viability is often compromised during the bioprinting process due to the wall-stress effect. Researchers have attempted to improve cell viability. Yeo et al. constructed hADSC-laden core-sheath structures, resulting in significantly higher cell viability compared to a general alginate-based cell-printing process (Yeo et al., 2016). Park et al. optimized the composition of alginate in bioink for bioprinting, which maintained better cell growth in the printed constructs (Park et al., 2017). Shi et al. concluded that cell migration and proliferation could be tuned by controlling alginate stiffness during bioprinting (Shi et al., 2017). Here, we prepared smooth muscle-induced hADSC microtissues and mixed them with alginate-gelatin bioink for bioprinting; cell viability and proliferation were investigated and reported. In this article, we provide an innovative way to induce hADSC smooth muscle differentiation and preserve cell viability for bioprinting, which holds great promise for 3D bioprinting to construct native tissues involving urethra or vessels.

## Materials and methods

### Source of human adipose derived stem cells

Human adipose tissue samples were obtained from the abdomen of healthy females, and informed consent was approved by the institutional review boards. The collected samples were sliced, treated with 0.1% collagenase type I (Sigma-Aldrich, USA) under agitation for 90 min at 37°C, filtered through a 100 μm mesh, and centrifuged at 3,000 × g for 10 min. The supernatant was discarded, and the precipitate was resuspended with α-MEM (Corning) containing 10% fetal bovine serum (FBS, Corning) and 100 U/ml penicillin/streptomycin (Keygen, China). Finally, the cell suspension was seeded onto the 10 cm Petri dishes, then maintained over 4–5 days until confluence was reached, which was defined as passage 0. When cells reached 80–90% confluence, they were passaged by 0.25% EDTA-trypsin (Solarbio, China).

### Flow cytometry analysis of hADSCs

Flow cytometry was employed to characterize hADSCs. Cells at passage 3 were digested with 0.25% EDTA-trypsin and washed thrice in PBS after centrifugation (1,500 × g for 5 min). 1 × 10^4^ cells/tube were incubated in the dark at room temperature for 20 min with the following antibodies: CD44 FITC, CD105 APC, CD45 PE, CD34 PE (all purchased from BD Bioscience). Then, the cells were washed again twice with PBS, centrifuged (1,500 × g, 5 min) and resuspended in 200 μl PBS. Finally, CD surface antigens were analyzed with FlowCytometer (BD FACSCalibur, USA).

### Preparation of hADSCs microtissues and smooth muscle differentiation

Passages 3–5 of hADSCs were used to form MTs. Briefly, the hADSCs were detached from the Petri dishes and centrifuged. Then, the pellet was resuspended with culture medium (α-MEM containing 10% FBS and 100 U/ml penicillin/streptomycin) for internal inspection and, smooth muscle inductive medium (SMIM, α-MEM containing 10% FBS and 100 U/ml penicillin/streptomycin and 10 ng/ml TGF-β1 [Sigma-Aldrich]) for smooth muscle differentiation. The cell suspension was used to generate MTs via the hanging-drop method. Briefly, 5 × 10^5^ hADSCs were suspended in 1 ml of culture medium or SMIM and pipetted onto an inverted Petri dish lid with 20 μl of cell suspension per drop using a multi-channel pipette. Then, the lid was turned over and placed on a Petri dish containing 3 ml of PBS to reduce the evaporation of the droplets. The MTs were allowed to form based on gravity-enforced cell assembly. The MTs (10,000 cells in 20 μL) were kept for 3 days in a hanging-drop array at 37°C in a humidified atmosphere of 5% CO2. The P3 hADSCs maintained in Petri dishes were cultured in SMIM and termed the control group.

### Inspection of MT extracellular matrix and identification of smooth muscle differentiation

On the third day, immunofluorescence was used to inspect the internal cell-to-cell interactions and identify the smooth muscle differentiation of MTs and control cells. To inspect internal cell-to-cell interactions, MTs were collected and washed with phosphate buffer saline (PBS, Keygen, China) thrice, then subjected to frozen section for immunofluorescence. Briefly, the washed MTs were embedded in optimal cutting temperature compound (OCT, Sakura) and placed in a −80°C refrigerator for 10 min. Then, we prepared MT frozen sections with a thickness of 5 μm using a frozen microtome (Leica CM1950, Heidelberg, Germany). For the identification of smooth muscle differentiation, MTs were first collected and resuspended in SMIM, then seeded onto a Petri dish for 6 h for adhesion and subjected to immunofluorescence. After fixation with 4% paraformaldehyde for 20 min at room temperature, frozen MT sections, adherent MTs and control cells were washed three times with PBS. The samples were then permeabilized and blocked with 0.1% Triton X-100 and 5% goat serum (ZSGB-BIO, China) in PBS for 15 min at room temperature. Subsequently, the samples were incubated with rabbit anti-collagen IV (1:100, Abcam), rabbit anti-alpha smooth muscle Actin (1:200 Abcam), and mouse anti-smoothelin (1:200 Abcam) overnight at 4°C. Afterwards, the samples were incubated with FITC-conjugated goat anti-rabbit IgG, Cy3-conjugated goat anti-rabbit IgG, and FITC-conjugated goat anti-mouse IgG (1:1,000 Abcam) for 60 min at room temperature and rinsed with PBS thrice. For nuclear staining, the samples were incubated with 4′,6-diamidino-2-phenylindole (DAPI, Keygen, China) for 15 min. Lastly, the samples were photographed and recorded using a LEICA DMI 4000B digital microscope camera system (Leica, Heidelberg, Germany).

### Western blotting

Smooth muscle markers were tested in MTs and control cells after the transfer to new adherent plastic Petri dishes. Briefly, cells were washed with 4°C PBS thrice and then lysed with radio immunoprecipitation assay (RIPA, ApplyGen Technology, China) to obtain the total proteins. The concentration of total protein was measured with BCA Protein Quantitation Assay (KeyGEN, China). Forty micrograms of protein were then separated by 8% sodium dodecyl sulfate-polyacrylamide gel electrophoresis (SDS-PAGE) and transferred to polyvinylidene fluoride (PVDF, Millipore) membranes. The membranes were blocked with 5% non-fat dry milk (Cell Signaling) in Tris-Buffered Saline Tween-20 (TBST, ApplyGen Technology, China) buffer for 2 h. Then, the membranes were incubated with primary antibodies at 4°C overnight. The primary antibodies were as follows: rabbit polyclonal to alpha smooth muscle actin (1:1,000 Abcam) and mouse monoclonal to smoothelin (1:1,000 Abcam). Next, the membranes were washed with TBST for 10 min three times. Then, the membranes were incubated with HRP-conjugated anti-rabbit and anti-mouse IgG secondary antibodies (1:5,000 ZSGB-BIO, China) for 90 min at room temperature and washed thrice with TBST. After adding enhanced chemiluminescence detection regents (ApplyGen Technology, China), the membranes were visualized by scanning the immunostaining band (GE ImageQuant LAS4000). The band intensity was analyzed with Image-Pro-plus 6.0 software.

### 3D bioprinting

Both cells from the MTs and cells from the control group were suspended in bioink at a concentration of 10^6^ cells/ml and then subjected to 3D bioprinting using a printing platform (Regenovo 3D bioprinter, China). Bioink was prepared as follows: Briefly, cells were embedded into hydrogels using a 20 ml hyperthermia-dissolved solution of 0.2 g/ml gelatin (Sigma-Aldrich) mixed evenly with a 10 ml solution including 0.04 g/ml sodium alginates (Sigma-Aldrich) at 25°C. We used a syringe with an orifice diameter of 340 μm for preparing the tissue-printed construct. Before the bioprinting process, all used syringes, syringe-paired inner sealers, and nozzles were autoclaved and stored under aseptic conditions at 4°C. The synthetic bioink of the MTs and the control cells was maintained in an identical sterilized chamber at 4°C for 20 min to keep it gelated. Then, the bioprinting process was performed. First, the printing platform with a temporal-fixed 60 mm Petri dish used as a substrate was rapidly cooled for 45 min to 4°C and underwent ultraviolet sterilization. Then, we fabricated the desired 3D structure by maintaining the bioink at T <8°C during extrusion from the syringe and placement. Lastly, each fresh 3D structure was immediately sprinkled with 2 ml of sterile 10% calcium chloride at 4°C for 10 min to complete the cross-linking process, then immersed in SMIM and cultured in the incubator at 37°C in a humidified atmosphere of 5% CO2.

### Characterization of bioprinted 3D structure

We utilized HE and immunofluorescence staining to examine the morphology and stability of the induced MTs in the bioprinted 3D structure. After the bioprinting process was finished, the 3D structure was embedded in optimal cutting temperature compound (OCT, Sakura) and subjected to frozen sectioning for HE and immunofluorescence staining. For HE staining, frozen sections were washed briefly in distilled water, stained in hematoxylin solution (Beyotime Biotechnology, China) for 5 min, washed in running tap water for 1 min, differentiated in 1% acid alcohol for 6 s, washed in running tap water again for 1 min, blued in 0.2% ammonia water, washed in running tap water for 1 min, counterstained in eosin solution (Beyotime Biotechnology, China) for 5 min, dehydrated in alcohol (75% × 2 for 2 s each, 80% × 2 for 2 s each, 95% × 2 for 1 min each, 100% × 2 for 1 min each), and cleared in 2 changes of xylene for 3 min each. For immunofluorescence staining, we followed the protocol above. Samples were photographed and recorded using a LEICA DMI 4000B digital microscope camera system (Leica, Heidelberg, Germany).

### Cell viability and proliferation assay

Cell viability was determined by a live/dead test, and the metabolic activity of cells within the 3D structure was determined by a cell count kit 8 (CCK-8, Beyotime, China). For the live/dead test, we used the Live Dead Viability/Cytotoxicity kit (Life Technologies). Briefly, the 3D structure was exposed to 0.15 mM calcein-AM and 2 mM ethidium homodimer-1 for 40 min in a lucifuge box at room temperature. The stained structure was then photographed using the LEICA DMI 4000B digital microscope camera mentioned above. To evaluate the cell viability at different time points, the numbers of live and dead cells were counted using the Image-Pro-plus 6.0 software, and the ratio of the number of live cells to the number of total cells was calculated.

For the CCK-8 test, 1 ml of the 3D structures was immersed in 400 μl of CCK-8 solution and incubated for 2 h at 37°C. Then, 20 μl of the supernatant were transferred to a 96-well plate, and the absorbance was measured at 450 nm using a microplate reader. Three-dimensional structure samples were not recycled, different samples were prepared for different time points, and each test was performed in triplicate.

### Statistical analysis

Statistical analyses were performed with SPSS 17.0 software (SPSS Inc., Chicago, IL, USA). All data are presented as the mean ± standard deviation (SD). The between group comparison was done using the *t*-test, and *P* < 0.05 was considered statistically significant.

## Results

### Identification of hADSCs

**Figure 1:**
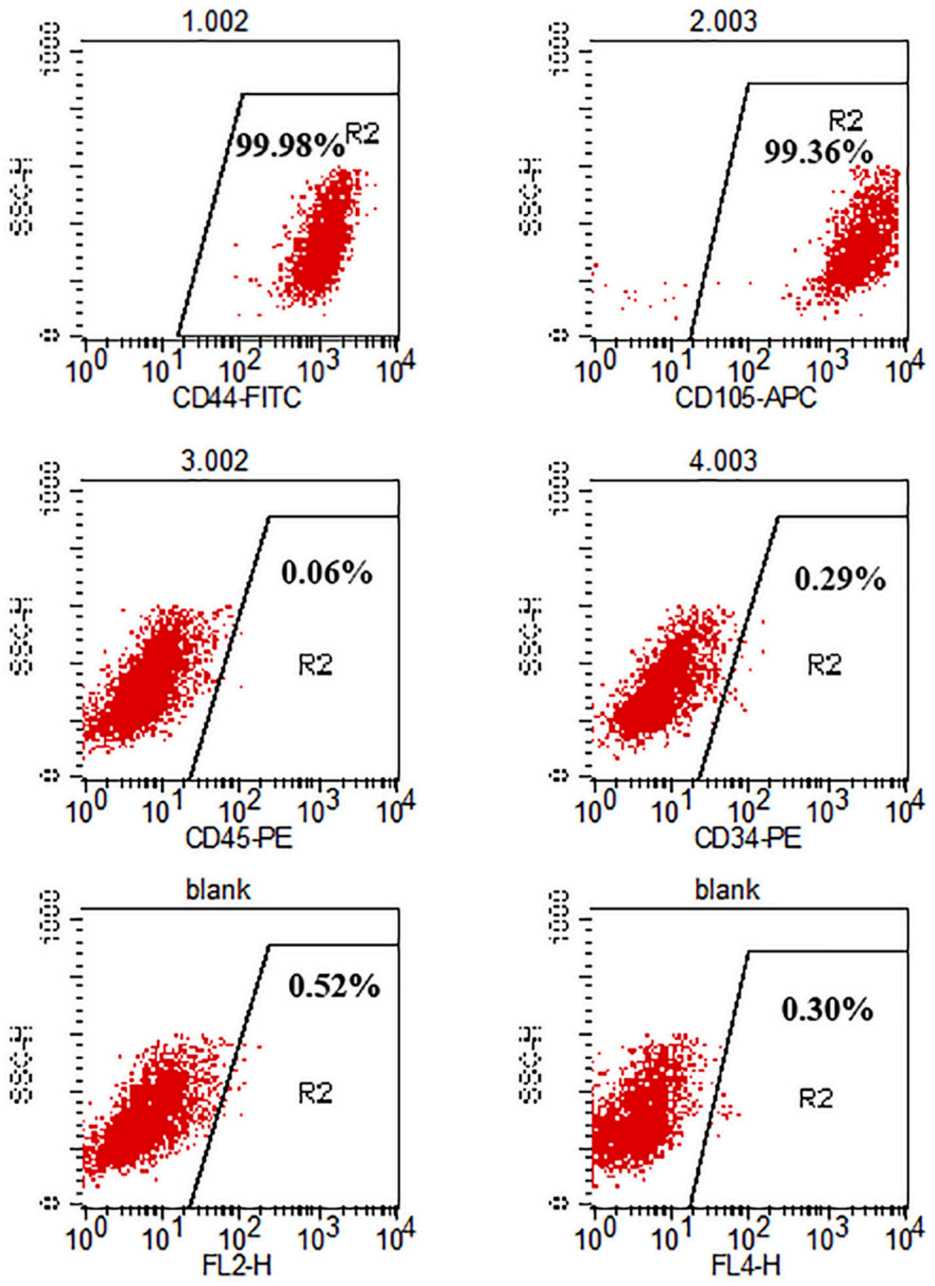
Flow cytometry analysis of hADSC surface phenotype. CD44 and CD105 were positive while CD45 and CD34 were negative. The percentage of positive cells is indicated in the figure.

The cell surface antigens of the hADSCs were assayed with flow cytometry. We selected typical surface proteins to identify the hADSCs. The results showed that hADSCs presented a strong positivity for CD44 and CD105, while no signal was detected for CD45 and CD34. The percentage of positive cells was 99.71, 99.50, 0.01, and 0.06%, respectively (Figure 1).

### Generation, adhesion, and inspection of MTs

**Figure 2:**
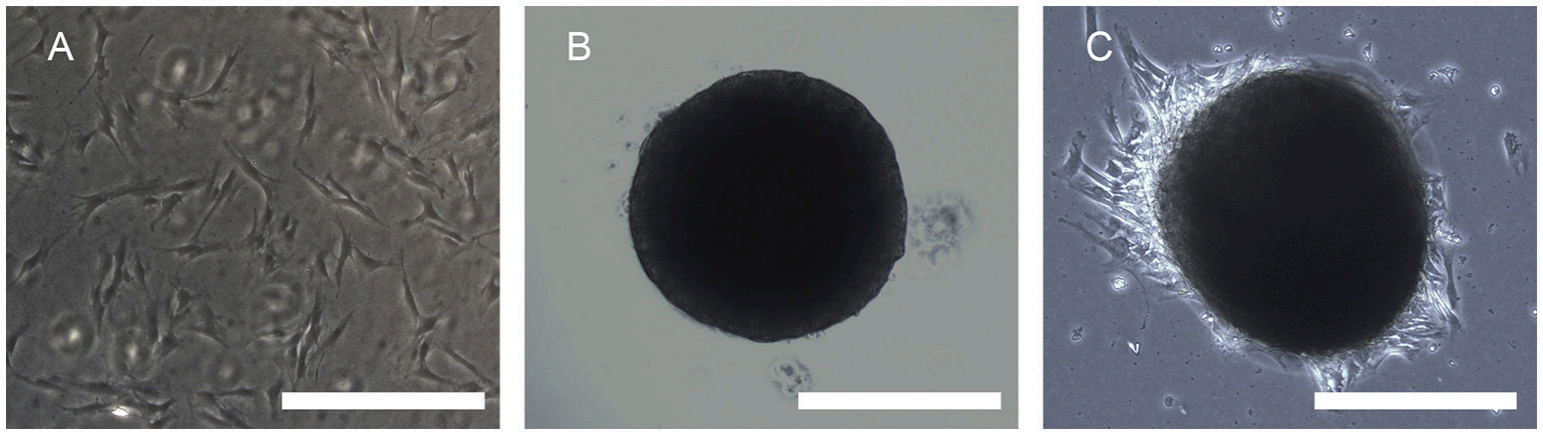
Phase contrast microscopy showing the aggregation process of 10,000 hADSCs into microtissues (MTs) and the post-adhesion MTs on a Petri dish. **(A)** hADSCs cultured on a Petri dish (passage 4); **(B)** morphology of MTs on day 3. The appearance of MTs became stable with a smooth edge on day 3; **(C)** MTs on day 3 seeded onto a Petri dish for 6 h for adhesion; cells were seen around the adherent MTs. Inset bar = 200 μm.

**Figure 3:**
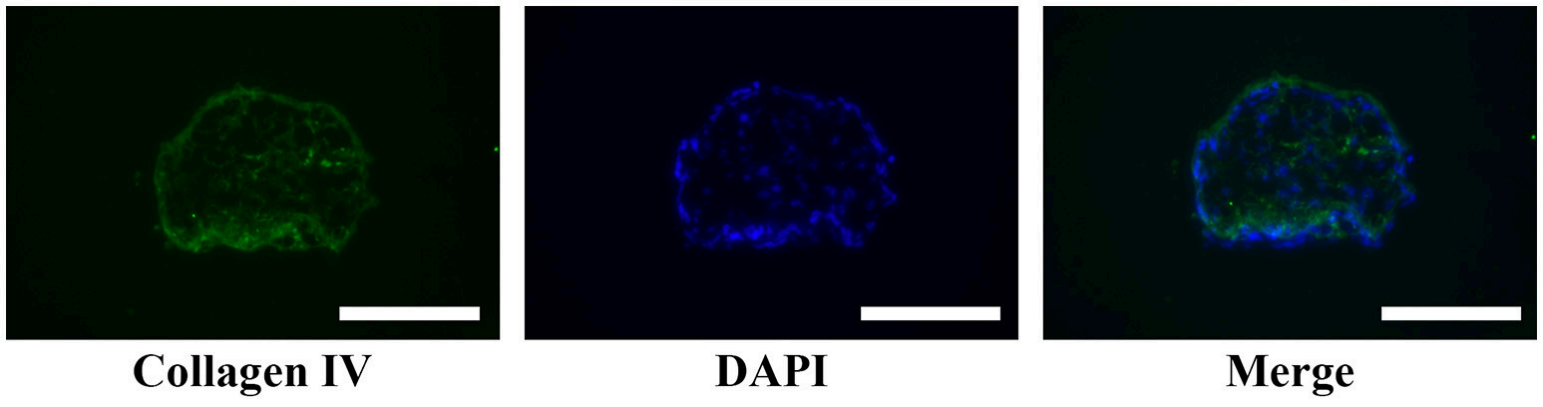
Immunofluorescence showed type IV collagen was positive in the internal part of MTs, reflecting the formation of extracellular matrix in MTs. Inserted bar = 200 μm.

A large number of hADSCs could be isolated by type I collagenase digestion from adipose tissue. After 4–5 days of isolation, primary hADSCs could adhere to the wall of the Petri dish and presented a “spindle-like” morphology (Figure 2A). Passage 3 of the hADSCs was obtained to perform the hanging-drop procedure to produce the MTs, and the MTs on days 1–3 were photographed and measured. The mean diameter was 252.18 ± 23.78, 236.2 ± 20.22, 216.79 ± 15.85, and 230.58 ± 17.51 μm on the first day, second day, third day, and post-adhesion, respectively. Figures 2B,C presented morphology of MTs at day 3 and post-adhension MTs. Immunofluorescence confirmed type IV collagen was positive in MTs (Figure 3), suggesting the generation of extracellular matrix (ECM).

### Smooth muscle differentiation in hADSCs by immunofluorescence and western blotting assays

**Figure 4:**
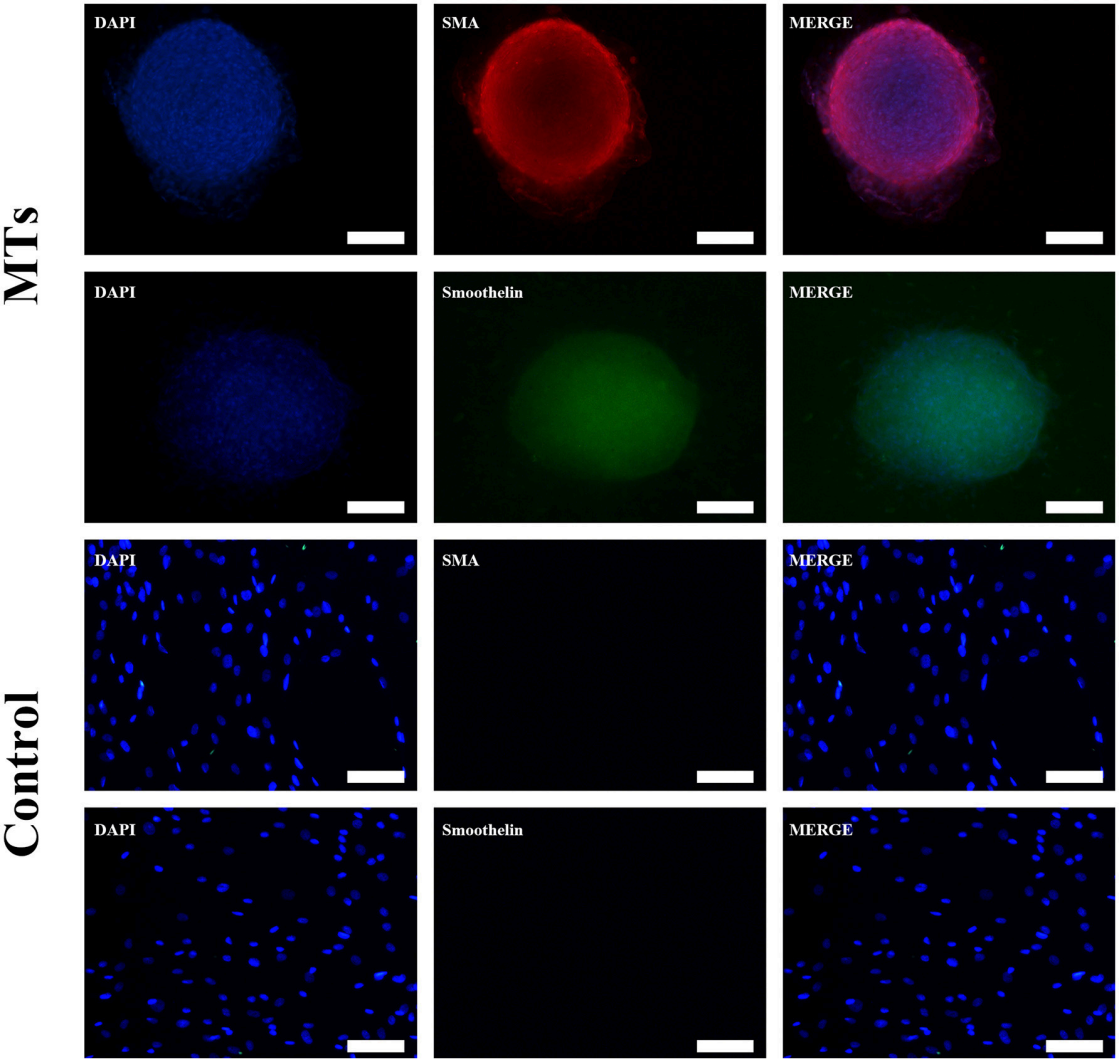
Smooth muscle differentiation of MTs and the control group. The immunofluorescence detected α-SMA and smoothelin expression in microtissues (MTs) and hADSCs cultured in a Petri dish (Control). Inset bar = 100 μm.

After the MTs and the control cells were induced in smooth muscle inductive medium for 3 days, the immunofluorescence was positive for α-smooth muscle actin and smoothelin in MTs but negative in control cells (Figure 4). These results suggest that hADSCs could be successfully induced into smooth muscle-like cells by the hanging-drop method over a relatively short period of time (3 days).

**Figure 5:**
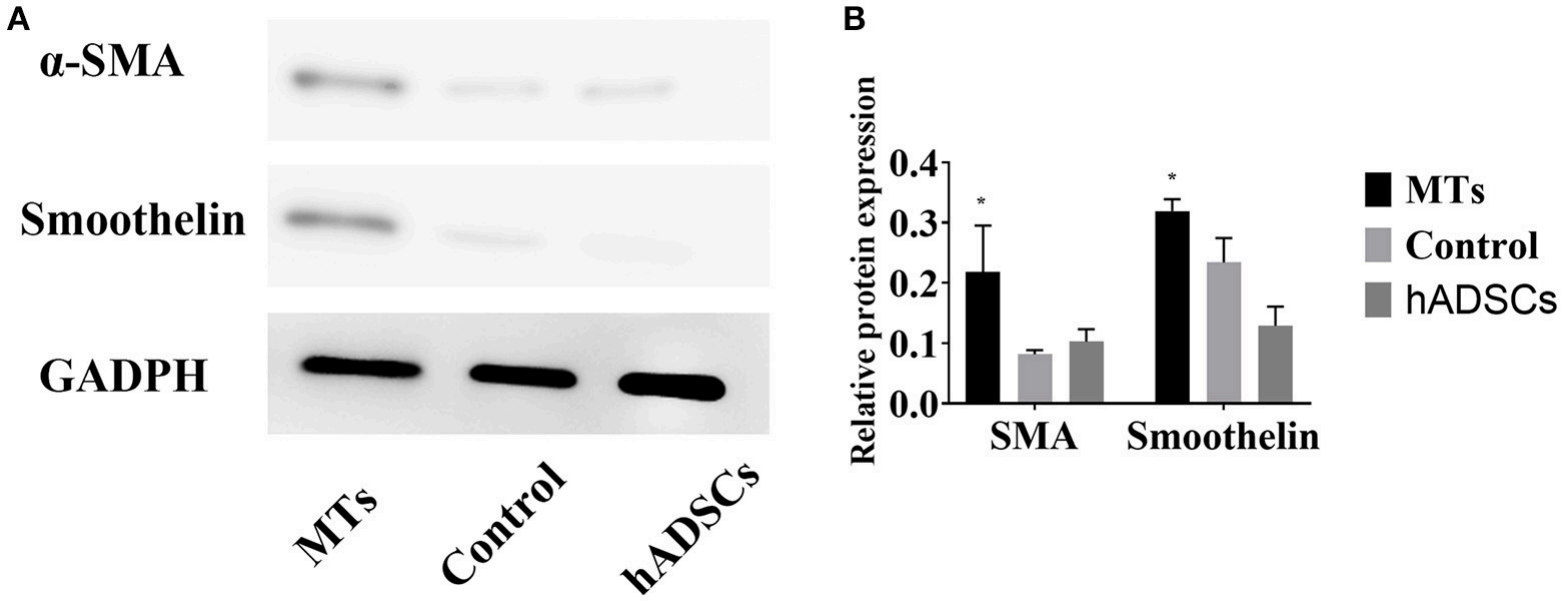
**(A)** western blotting compared the relative protein expression of α-SMA and smoothelin in microtissues (MTs) and induced monolayer cultured hADSCs (control) and non-induced hADSCs (hADSCs); **(B)** Semi-quantitative data of relative protein expression (target protein/GADPH). ^*^P < 0.05 vs. the control and hADSCs.

To further investigate differences in smooth muscle differentiation between the MTs and the control cells, western blotting was employed to detect the relative protein expression levels of α-SMA and smoothelin. The result of western blotting showed that the relative protein expression levels of α-SMA and smoothelin were higher in the MTs than in the control cells (Figures 5A,B). These results suggested that MTs formed by the hanging-drop method could improve the smooth muscle differentiation potential of hADSCs compared to hADSCs induced on Petri dishes. Control antibody conditions were utilized in immunofluorescence of collagen IV, α-SMA and Smoothelin, the results is showed in the supplementary figures accordingly (Supplementary Figures 1, 2).

### Gross and optical observations of MTs and control cells in the 3D bioprinted structure

**Figure 6:**
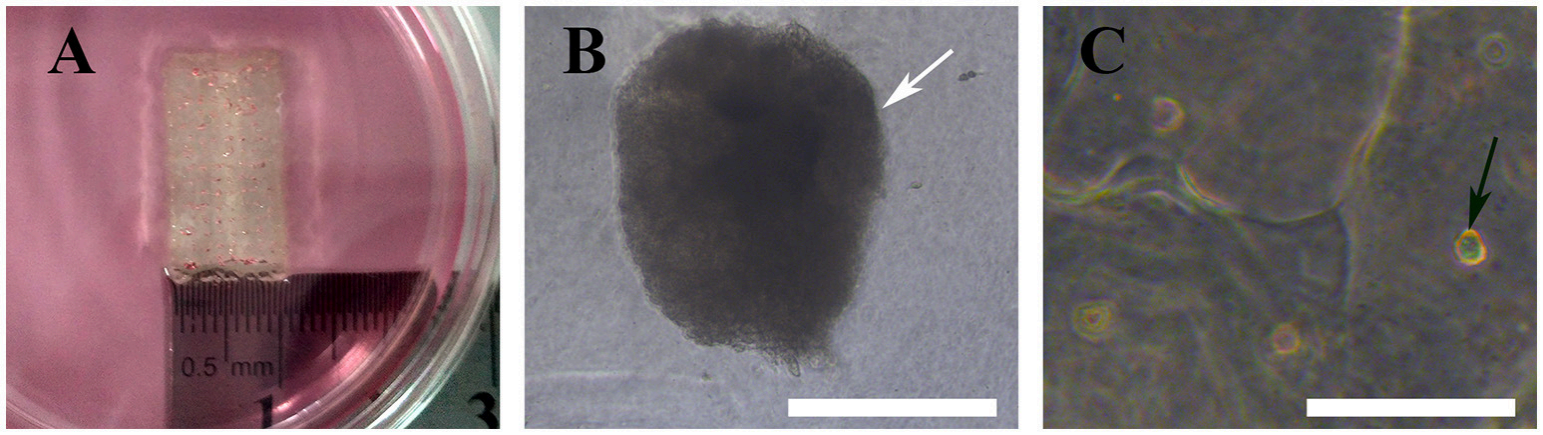
Bioprinted 3D structure immersed in culture medium **(A)**, optical observation of MTs (**B**, white arrow) and hADSCs (**C**, black arrow) in the bioprinted structure. Inset bar = 200 μm.

We bioprinted a 3D structure at 20 × 10 × 1 mm^3^ (length × width × height) and immersed it in α-MEM culture medium for gross and optical observation. As shown below, gross inspection revealed that the 3D bioprinted 3D maintained its configuration (Figure 6A) and MTs bioprinted onto the 3D structure maintained their shape and diameter (Figure 6B). HADSCs detached through monolayer culturing for bioprinting exhibited a round or oval shaped morphology in the 3D structure (Figure 6C).

### Morphology and stability of induced MTs and hADSCs in the 3D structure

**Figure 7:**
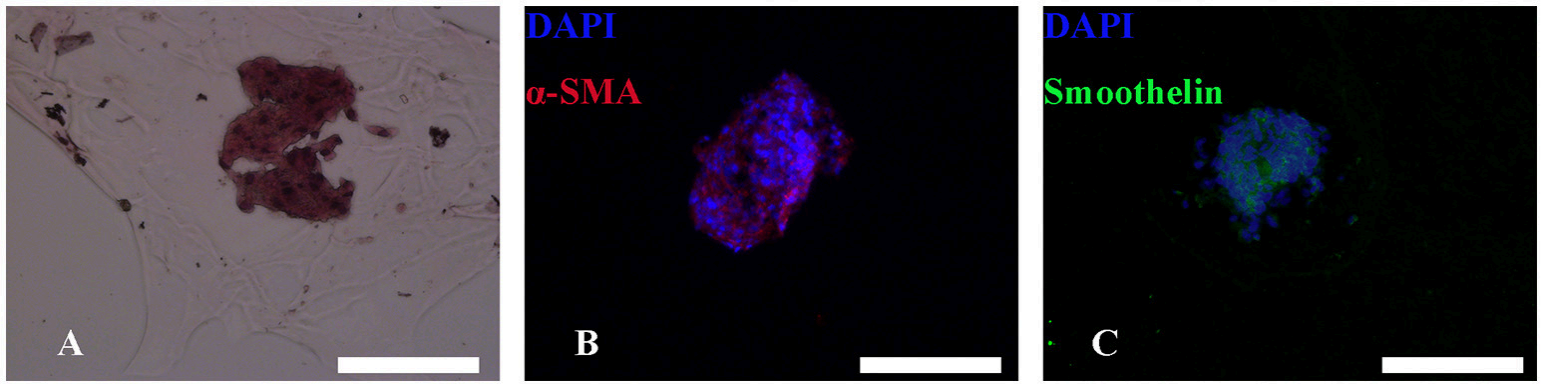
HE and immunofluorescence of MTs in the bioprinted 3D structure. HE staining revealed that MTs could largely sustain their morphology after the bioprinting process **(A)**. Immunofluorescence confirmed induced MTs still had expression of α-SMA **(B)** and smoothelin **(C)**. Red, α-SMA; green, smoothelin; blue, DAPI; inset bar = 200 μm.

After bioprinting, HE staining showed that induced MTs had largely maintained their morphology (Figure 7A), and induced MTs still expressed α-SMA and smoothelin in the 3D structure, which was confirmed with immunofluorescence (Figures 7B,C).

### Cell viability and proliferation assay using the live-dead and CCK-8 tests

**Figure 8:**
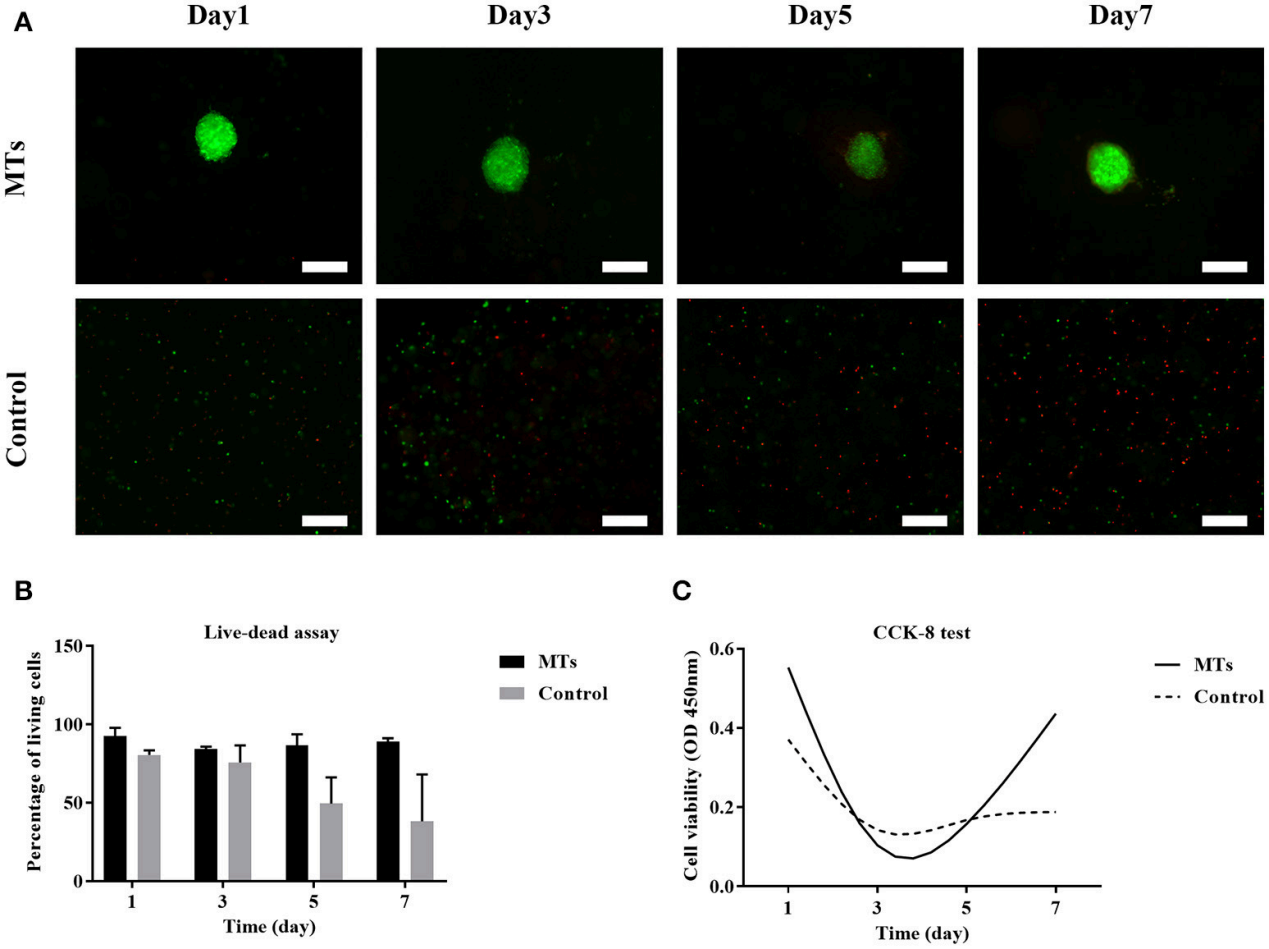
Live-dead assay of MTs and the control group bioprinted in the 3D structure **(A)**. Fluorescence image showed live cells (green) and dead cells (red), inset bar = 100 μm. The quantitative data of cell viability at different time points in the bioprinted 3D structure **(B)**. CCK-8 test to detect the proliferation of MTs and the control group in the bioprinted 3D structure **(C)**.

The live-dead test was performed and the percentage of live cells was calculated to evaluate cell viability. For MTs, the cell viability was 92.6 ± 5.18, 84.35 ± 1.5, 86.56 ± 7.15, and 89.12 ± 2.1% on day 1, day 3, day 5, and day 7, respectively. In the control group, cell viability was 83.62 ± 2.78, 75.69 ± 10.81, 49.5 ± 16.63, and 38.22 ± 29.84 on day 1, day 3, day 5, and day 7, respectively (Figures 8A,B). The results from the CCK-8 assay showed that both MTs and hADSCs were proliferating in the bioprinted 3D structure. For the MTs and the control cells bioprinted onto the 3D structure, the CCK-8 assay absorption values (reading of culture media absorption at 450 nm) were 0.55 ± 0.02 and 0.37 ± 0.04 on day 1 and decreased to 0.11 ± 0.1 and 0.14 ± 0.05 on day 3, which indicated that the pressure-driven bioprinting process may have had a negative effect on the cells in the 3D structure. On day 5 and day 7, the CCK-8 assay absorption values drastically increased to 0.16 ± 0.09 and 0.44 ± 0.07 for MTs and slightly increased to 0.16 ± 0.04 and 0.19 ± 0.06, respectively, in the control group (Figure 8C), suggesting that proliferation was more active in the MTs than in the control cells as time passed.

## Discussion

Human adipose derived stem cells (hADSCs) have gained popularity in tissue engineering studies many have achieved promising results in the reconstruction of various tissue defects. It is widely believed that hADSCs originated from the mesoderm. Based on this belief, hADSCs should be multipotent, allowing them to differentiate into myocytes, adipocytes, chondrocytes, and osteoblasts. Many studies have investigated various methods of smooth muscle differentiation in hADSCs, including the use of inducing factors, TIPS, and co-culturing with myoblasts (Di Rocco et al., 2006; Sakuma et al., 2009; Wang et al., 2010; Parmar et al., 2015). Though these methods induce hADSCs to differentiate into smooth muscle cells, some drawbacks should not be neglected: a long inducing period (3–6 weeks), the requirement of myoblasts for co-culturing, the requirement of equipment to fabricate microcarriers, etc. which limited its further application in cell therapies.

We noticed that some studies showed that spheroid cultures can enhance stemness and the therapeutic potential of hADSCs (Emmert et al., 2013; Bogdanova-Jatniece et al., 2014). We hypothesized that spheroid cultures may contribute to smooth muscle differentiation in the presence of smooth muscle inductive medium.

Currently, various spheroid culture methods have emerged to form cell aggregates and study their effect on MSCs, including the use of non-adherent plates or microfabrication-based non-adhesive surfaces, the hanging-drop method, spinner or rotary dynamic culture systems, or the use of agarose multi-well dishes from rubber micro-molds (Song et al., 2004; Nyberg et al., 2005; Bogdanova-Jatniece et al., 2014; Rettinger et al., 2014; Guo et al., 2015; Nakagawa et al., 2016). Compared to other methods, the hanging-drop method is relatively economical and convenient. Emmert et al. generated MTs from different human stem cells (human bone marrow and adipose tissue-derived MSCs, Isl1^+^ cardiac progenitor cells derived from human embryonic stem cells, undifferentiated human induced pluripotent cells) via the hanging-drop method; their results suggested that these four cell lines can successfully generate MTs. The ECM distribution was homogenous for human adipose tissue-derived MSCs and undifferentiated human induced pluripotent cells, while it was primarily concentrated within the center of the other two cell lines (Emmert et al., 2013). Among the studies mentioned above, Nakagawa et al. aggregated human bone marrow MSCs using the hanging-drop method and transplanted the aggregates into osteochondral defects in rats. The outcome of this study was that aggregation promoted lubricin expression of MSCs *in vitro*, and the morphology of the superficial cartilage in the MSC group was closer to that of the intact cartilage in the rat osteochondral defect model (Nakagawa et al., 2016). These researchers proved that utilizing the hanging-drop method to aggregate MSCs is a useful tool for tissue engineering.

In this study, we combined the hanging-drop method and TGF-β1 to generate smooth muscle-like MTs from hADSCs. Immunofluorescence staining detected the expression of type IV collagen in non-induced MTs. The expression of smooth muscle cell markers such as, α-SMA and smoothelin induced by TGF-β1 in a relatively short time (3 days) revealed that expression was higher in MTs than in their counterpart hADSC monolayer; this finding was confirmed by western blotting assay. That is, spheroid culture could promote the smooth muscle differentiation of hADSCs induced by TGF-β1. Calabrese et al. fabricated a new Collagen I-based 3D scaffold and assessed its chondrogenic potential *in vitro*, which promoted the early chondrogenic differentiation of hADSCs (Calabrese et al., 2017). Guo et al. reported that spheroid formations may enhance the neural differentiation potential of post-thaw hADSCs (Guo et al., 2015). Other research has shown that hypoxia reinforces proliferation and VEGF expression of hADSCs (Lee et al., 2009) suggesting that the stemness of hADSCs might be intensified via a hypoxic environment in the inner parts of MTs. In our previous study, we proved VEGF expression is significantly increased in MTs compared to hADSCs without spheroid formations (Xu et al., 2014), which is consistent with the aforementioned studies.

Collagen IV is an extracellular matrix protein that forms heterotrimers and is present in nearly all basement membranes in every organ (Jeanne and Gould, 2017). Based on this finding, we assumed that it is the enhanced stemness of spheroid cultures that promotes the production of collagen IV, which is the main element of ECM, and provided a “microenvironment” for smooth muscle differentiation. Our results confirmed the findings of the aforementioned studies (Emmert et al., 2013). Furthermore, after the bioprinting process, induced MTs maintained their phenotype, suggesting that they might possess smooth muscle function in the 3D structure. Yao et al. encapsulated hADSCs in alginate and alginate/gelatin microspheres, and fabricated these microspheres via an microsphere generating device. Results suggested that compared with pure alginate, alginate/gelatin microspheres could achieve higher cell proliferation as well as adipogenic differentiation, which are similar to our findings. (Yao et al., 2012).

3D bioprinting sheds light on tissue engineering by providing a platform to make a computer designed construct of cells and bioinks, and a bioprinted construct might be a promising strategy in tissue regeneration. There are various types of 3D bioprinting, including stereolithography, bioplotting, and fused deposition modeling (O'Brien et al., 2015). Among these types, bioplotting, or so-called micro-extrusion bioprinting, are the major printing technologies that can print cell-laden constructs under physiological conditions. Although micro-extrusion bioprinting can achieve homogenous cell distribution within the construct, cell viability is usually compromised due to some negative factors during the printing process. These negative factors include increased dispensing pressure and printing time, decreased nozzle diameter and temperature, and decreased thermal sensitive bioink temperatures (Panwar and Tan, 2016). Many researchers investigated ways to achieve better cell viability. Tan et al. employed poly(D, L-lactic-co-glycolic acid; PLGA) to construct cell-laden porous microspheres for 3D printing and observed high cell viability up to 14 days (Tan et al., 2016). Park et al. prepared bioinks with high and low molecular weight alginate for 3D printing and suggested that high and low alginate at a 2:1 ratio produced the best cell growth (Park et al., 2017). Yeo et al. fabricated a core (cell-laden collagen)/sheath (pure alginate) structure using hADSCs as the laden cells. They reported that this core-sheath structure exhibited outstanding cell viability compared to an alginate-based mesh structure (Yeo et al., 2016). In our study, we generated MTs from hADSCs and formed them into a 3D structure using a micro-extrusion bioprinter. The results of live-dead staining and CCK-8 assay showed that cell viability and cell proliferation are more satisfactory in MTs than in bioprinted single hADSCs. Additionally, we noticed that after bioprinting, the live cell percentage at the edge of the MTs decreased with the passage of time, and the CCK-8 assay of bioprinted MTs presented a “U-like” curve. Three potential underlying mechanisms could explain this effect: 1, Wall shear stress during the bioprinting process, may have damaged the outer part of the MTs while the inner part of remained intact. Thus, cells in MTs proliferated in the following days. 2, The existence of type IV collagen in MTs provided an ECM for cell proliferation. 3, Compared to a single-cell bioprinting strategy, using MTs for bioprinting skipped the step of hADSC detachment from the Petri dish, which may handicap cell viability.

The other studies mentioned above were equipment-dependent or used single-composition bioink (alginate alone), which may harm the supportive ability of cells. Here, we changed the bioprinting strategy by using MTs instead of traditional single-cells and mixed MTs with alginate-gelatin bioink, which were easily achieved by a routine micro-extrusion bioprinter and maintained its cell supportive ability with the addition of gelatin.

In conclusion, our results showed that transforming hADSCs into MTs is a feasible way to enhance smooth muscle differentiation and maintain more robust cell viability and cell proliferation than conventional single-cell strategies for bioprinting up to 7 days. We speculated that the improved stemness of hADSCs by generating MTs had a positive effect on its smooth muscle differentiation, and it is the outer part that prevented the inner part of MTs from harmful shear stress during the bioprinting process. This work revealed that MTs consisting of hADSCs could play an important role in the construction of 3D structures and further applications in tissue engineering.

## Author contributions

The experimental sections were completed by JY and XY helped redesign the experiments and performed quality control procedures. JY drafted the manuscript. WY, SJ, and GuJ helped revise the manuscript. The corresponding authors, GaJ and YY designed the experiments, coordinated, and helped draft the manuscript. All authors have read and approved the final manuscript.

### Conflict of interest statement

The authors declare that the research was conducted in the absence of any commercial or financial relationships that could be construed as a potential conflict of interest.

## References

[B1] Bajek A. Gurtowska N. Olkowska J. Kazmierski L. Maj M. Drewa T. (2016). Adipose-derived stem cells as a tool in cell-based therapies. Arch. Immunol. Ther. Exp. 64, 443–454. 10.1007/s00005-016-0394-x 27178663 PMC5085986

[B2] Bogdanova-Jatniece A. Berzins U. Kozlovska T. (2014). Growth properties and pluripotency marker expression of spontaneously formed three-dimensional aggregates of human adipose-derived stem cells. Int. J. Stem Cells 7, 143–152. 10.15283/ijsc.2014.7.2.143 25473452 PMC4249897

[B3] Calabrese G. Forte S. Gulino R. Cefali F. Figallo E. Salvatorelli L.. (2017). Combination of collagen-based scaffold and bioactive factors induces adipose-derived mesenchymal stem cells chondrogenic differentiation *in vitro*. Front. Physiol. 8:50. 10.3389/fphys.2017.00050 28210226 PMC5288372

[B4] Choi Y. S. Matsuda K. Dusting G. J. Morrison W. A. Dilley R. J. (2010). Engineering cardiac tissue *in vivo* from human adipose-derived stem cells. Biomaterials 31, 2236–2242. 10.1016/j.biomaterials.2009.11.097 20031204

[B5] Di Rocco G. Iachininoto M. G. Tritarelli A. Straino S. Zacheo A. Germani A.. (2006). Myogenic potential of adipose-tissue-derived cells. J. Cell Sci. 119, 2945–2952. 10.1242/jcs.03029 16825428

[B6] Emmert M. Y. Wolint P. Wickboldt N. Gemayel G. Weber B. Brokopp C. E.. (2013). Human stem cell-based three-dimensional microtissues for advanced cardiac cell therapies. Biomaterials 34, 6339–6354. 10.1016/j.biomaterials.2013.04.034 23727259

[B7] Fu Q. Song X. F. Liao G. L. Deng C. L. Cui L. (2010). Myoblasts differentiated from adipose-derived stem cells to treat stress urinary incontinence. Urology 75, 718–723. 10.1016/j.urology.2009.10.003 19969332

[B8] Guo X. Li S. Ji Q. Lian R. Chen J. (2015). Enhanced viability and neural differential potential in poor post-thaw hADSCs by agarose multi-well dishes and spheroid culture. Hum. Cell 28, 175–189. 10.1007/s13577-015-0116-4 26054839

[B9] Jeanne M. Gould D. B. (2017). Genotype-phenotype correlations in pathology caused by collagen type IV alpha 1 and 2 mutations. Matrix Biol. 57–58, 29–44. 10.1016/j.matbio.2016.10.003 27794444 PMC5328961

[B10] Lee E. Y. Xia Y. Kim W.-S. Kim M. H. Kim T. H. Kim K. J.. (2009). Hypoxia-enhanced wound-healing function of adipose-derived stem cells: increase in stem cell proliferation and up-regulation of VEGF and bFGF. Wound Repair Regen. 17, 540–547. 10.1111/j.1524-475X.2009.00499.x 19614919

[B11] Meligy F. Y. Shigemura K. Behnsawy H. M. Fujisawa M. Kawabata M. Shirakawa T. (2012). The efficiency of *in vitro* isolation and myogenic differentiation of MSCs derived from adipose connective tissue, bone marrow, and skeletal muscle tissue. In Vitro Cell. Dev. Biol. Anim. 48, 203–215. 10.1007/s11626-012-9488-x 22396125

[B12] Mineda K. Feng J. Ishimine H. Takada H. Doi K. Kuno S.. (2015). Therapeutic potential of human adipose-derived stem/stromal cell microspheroids prepared by three-dimensional culture in non-cross-linked hyaluronic acid gel. Stem Cells Transl. Med. 4, 1511–1522. 10.5966/sctm.2015-0037 26494781 PMC4675504

[B13] Nakagawa Y. Muneta T. Otabe K. Ozeki N. Mizuno M. Udo M.. (2016). Cartilage derived from bone marrow mesenchymal stem cells expresses lubricin *in vitro* and *in vivo*. PLoS ONE 11:e0148777. 10.1371/journal.pone.0148777 26867127 PMC4750963

[B14] Nyberg S. L. Hardin J. Amiot B. Argikar U. A. Remmel R. P. Rinaldo P. (2005). Rapid, large-scale formation of porcine hepatocyte spheroids in a novel spheroid reservoir bioartificial liver. Liver Transpl. 11, 901–910. 10.1002/lt.20446 16035089

[B15] O'Brien C. M. Holmes B. Faucett S. Zhang L. G. (2015). Three-dimensional printing of nanomaterial scaffolds for complex tissue regeneration. Tissue Eng. Part B Rev. 21, 103–114. 10.1089/ten.teb.2014.0168 25084122 PMC4322091

[B16] Panwar A. Tan L. P. (2016). Current status of bioinks for micro-extrusion-based 3D bioprinting. Molecules 21:E685. 10.3390/molecules21060685 27231892 PMC6273655

[B17] Park J. Lee S. J. Chung S. Lee J. H. Kim W. D. Lee J. Y.. (2017). Cell-laden 3D bioprinting hydrogel matrix depending on different compositions for soft tissue engineering: characterization and evaluation. Mater. Sci. Eng. C Mater. Biol. Appl. 71, 678–684. 10.1016/j.msec.2016.10.069 27987760

[B18] Park W. S. Heo S. C. Jeon E. S. Hong Da H. Son Y. K. Ko J. H.. (2013). Functional expression of smooth muscle-specific ion channels in TGF-β(1)-treated human adipose-derived mesenchymal stem cells. Am. J. Physiol. Cell Physiol. 305, C377–C391. 10.1152/ajpcell.00404.2012 23761629 PMC3891216

[B19] Parmar N. Ahmadi R. Day R. M. (2015). A novel method for differentiation of human mesenchymal stem cells into smooth muscle-like cells on clinically deliverable thermally induced phase separation microspheres. Tissue Eng. Part C Methods 21, 404–412. 10.1089/ten.tec.2014.0431 25205072 PMC4382826

[B20] Rettinger C. L. Fourcaudot A. B. Hong S. J. Mustoe T. A. Hale R. G. Leung K. P. (2014). *In vitro* characterization of scaffold-free three-dimensional mesenchymal stem cell aggregates. Cell Tissue Res. 358, 395–405. 10.1007/s00441-014-1939-0 25012521

[B21] Rodriguez L. V. Alfonso Z. Zhang R. Leung J. Wu B. Ignarro L. J. (2006). Clonogenic multipotent stem cells in human adipose tissue differentiate into functional smooth muscle cells. Proc. Natl. Acad. Sci. U.S.A. 103, 12167–12172. 10.1073/pnas.0604850103 16880387 PMC1567713

[B22] Sakuma T. Matsumoto T. Kano K. Fukuda N. Obinata D. Yamaguchi K.. (2009). Mature, adipocyte derived, dedifferentiated fat cells can differentiate into smooth muscle-like cells and contribute to bladder tissue regeneration. J. Urol. 182, 355–365. 10.1016/j.juro.2009.02.103 19457498

[B23] Salemi S. Tremp M. Plock J. A. Andersson K. E. Gobet R. Sulser T.. (2015). Differentiated adipose-derived stem cells for bladder bioengineering. Scand. J. Urol. 49, 407–414. 10.3109/21681805.2015.1004642 25652651

[B24] Shearier E. Xing Q. Qian Z. Zhao F. (2016). Physiologically low oxygen enhances biomolecule production and stemness of mesenchymal stem cell spheroids. Tissue Eng. Part C Methods 22, 360–369. 10.1089/ten.tec.2015.0465 26830500 PMC4827318

[B25] Shi P. Laude A. Yeong W. Y. (2017). Investigation of cell viability and morphology in 3D bio-printed alginate constructs with tunable stiffness. J. Biomed. Mater. Res. A 105, 1009–1018. 10.1002/jbm.a.35971 27935198

[B26] Song H. David O. Clejan S. Giordano C. L. Pappas-Lebeau H. Xu L.. (2004). Spatial composition of prostate cancer spheroids in mixed and static cultures. Tissue Eng. 10, 1266–1276. 10.1089/ten.2004.10.1266 15363181

[B27] Tan Y. J. Tan X. Yeong W. Y. Tor S. B. (2016). Hybrid microscaffold-based 3D bioprinting of multi-cellular constructs with high compressive strength: a new biofabrication strategy. Sci. Rep. 6:39140. 10.1038/srep39140 27966623 PMC5155425

[B28] Wang C. Yin S. Cen L. Liu Q. Liu W. Cao Y.. (2010). Differentiation of adipose-derived stem cells into contractile smooth muscle cells induced by transforming growth factor-β1 and bone morphogenetic protein-4. Tissue Eng. Part A 16, 1201–1213. 10.1089/ten.tea.2009.0303 19895205

[B29] Xu Y. Guan R. Lei H. Li H. Wang L. Gao Z.. (2014). Therapeutic potential of adipose-derived stem cells-based micro-tissues in a rat model of postprostatectomy erectile dysfunction. J. Sex. Med. 11, 2439–2448. 10.1111/jsm.12636 25042722

[B30] Yao R. Zhang R. Luan J. Lin F. (2012). Alginate and alginate/gelatin microspheres for human adipose-derived stem cell encapsulation and differentiation. Biofabrication 4:025007. 10.1088/1758-5082/4/2/025007 22556122

[B31] Yeo M. Lee J.-S. Chun W. Kim G. H. (2016). An innovative collagen-based cell-printing method for obtaining human adipose stem cell-laden structures consisting of core–sheath structures for tissue engineering. Biomacromolecules 17, 1365–1375. 10.1021/acs.biomac.5b01764 26998966

