# Corrigendum: Microtissues Enhance Smooth Muscle Differentiation and Cell Viability of hADSCs for Three Dimensional Bioprinting

**DOI:** 10.3389/fphys.2017.00703

**Published:** 2017-09-11

**Authors:** Yipeng Jin, Yongde Xu, Yuanyi Wu, Jilei Sun, Jiaxiang Guo, Jiangping Gao, Yong Yang

**Affiliations:** ^1^Department of Urology, Chinese PLA General Hospital Beijing, China; ^2^Department of Urology, First Affiliated Hospital of Chinese PLA General Hospital Beijing, China

**Keywords:** human adipose derived stem cells, microtissues, smooth muscle differentiation, 3D bioprinting, tissue engineering, cell viability

All authors' name were incorrectly spelled as [Jin Yipeng, Xu Yongde, Wu Yuanyi, Sun Jilei, Guo Jiaxiang, Gao Jiangping and Yang Yong]. The correct spelling is [Yipeng Jin, Yongde Xu, Yuanyi Wu, Jilei Sun, Jiaxiang Guo, Jiangping Gao and Yong Yang]. The authors apologize for this error and state that this does not change the scientific conclusions of the article in any way.

## Conflict of interest statement

The authors declare that the research was conducted in the absence of any commercial or financial relationships that could be construed as a potential conflict of interest.

