# Supplementary material for: Microtissues Enhance Smooth Muscle Differentiation and Cell Viability of hADSCs for Three Dimensional Bioprinting
Source: Front Physiol. 2017 Jul 25;8:534. doi: 10.3389/fphys.2017.00534 (PMC5524823; doi:10.3389/fphys.2017.00534)
Supplement: Supplementary file 1 [file DataSheet1.DOCX]

Supplementary Material

Microtissues enhance smooth muscle differentiation of hADSCs and cell viability for three dimensional bioprinting

Yipeng Jin*, Yongde Xu

*** Correspondence:** Jiangping Gao E-mail: jpgao@163.com

# Supplementary Figures and Tables

For more information on Supplementary Material and for details on the different file types accepted, please see [here](http://home.frontiersin.org/about/author-guidelines#SupplementaryMaterial).

## Supplementary Figures
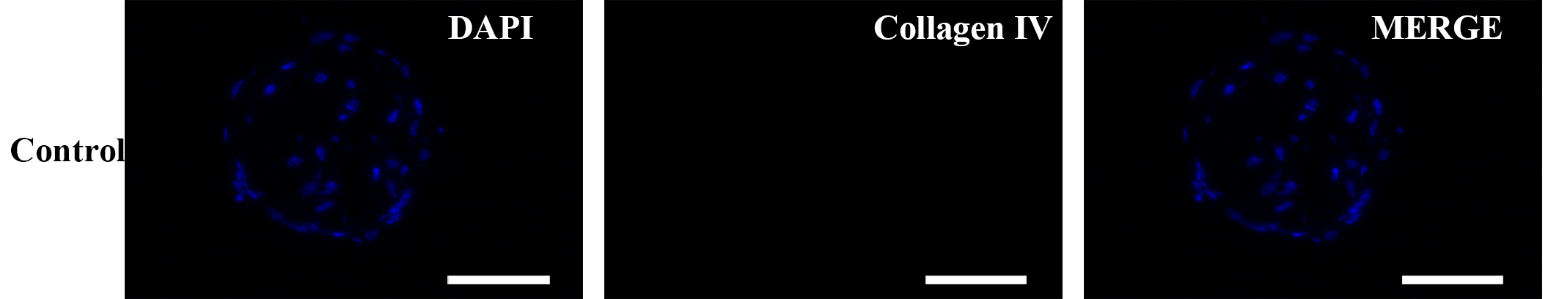


**Supplementary Figure 1.** Control antibody conditions for demonstrating specifity of collagen IV immunofluorescence staining.


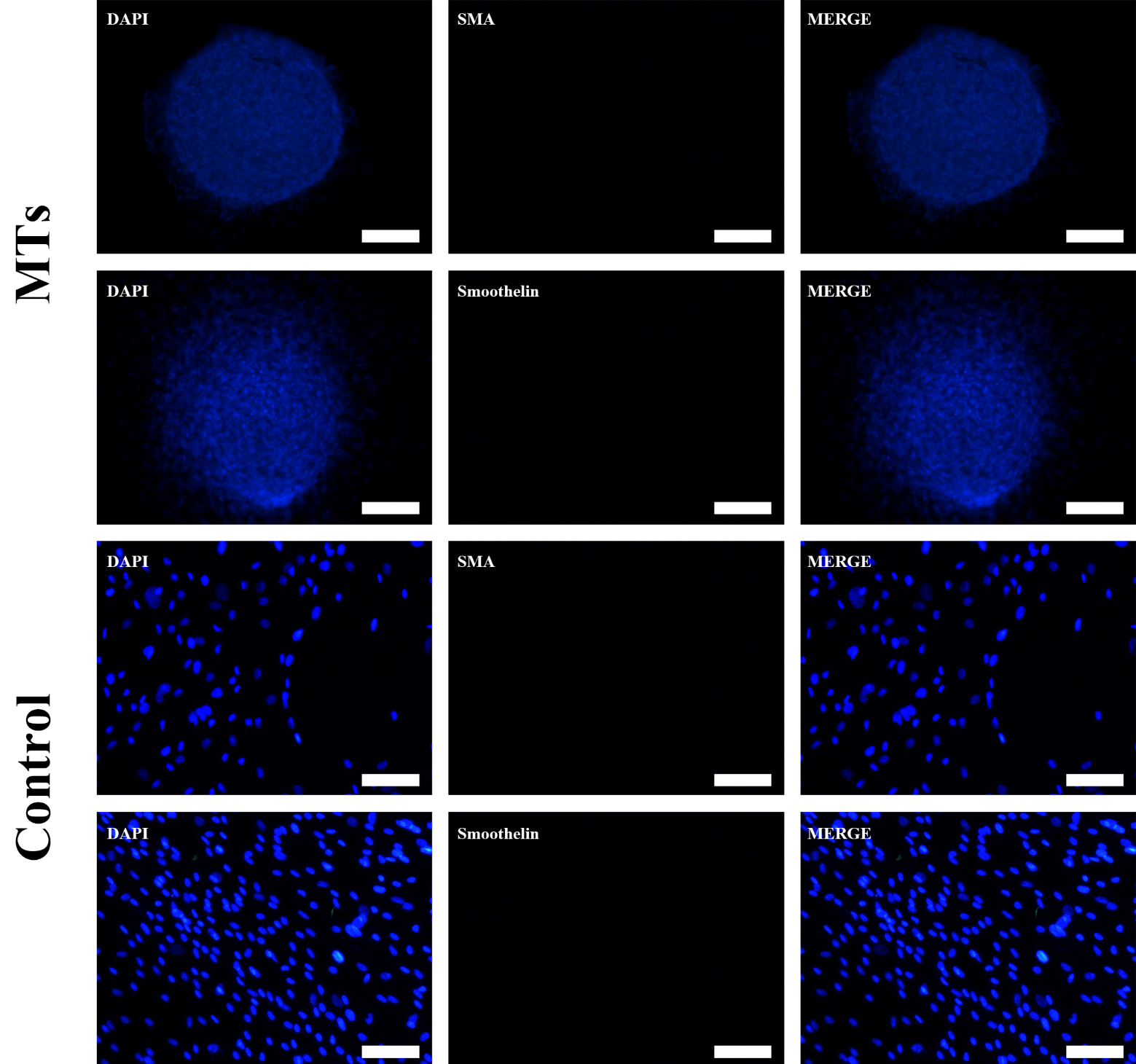


**Supplementary Figure 2.** Control antibody conditions for demonstrating specifity of α-SMA and Smoothelin immunofluorescence staining.
